# Cholinesterase Research

**DOI:** 10.3390/biom11081121

**Published:** 2021-07-30

**Authors:** Jan Korabecny, Ondrej Soukup

**Affiliations:** 1 Biomedical Research Center, University Hospital Hradec Kralove, Sokolska 581, 500 05 Hradec Kralove, Czech Republic; 2 Faculty of Military Health Sciences, University of Defence, Trebesska 1575, 500 05 Hradec Kralove, Czech Republic

Cholinesterases are fundamental players in the peripheral and central nervous systems. These serine hydrolases are presented by a two-membered family, namely acetylcholinesterase (AChE, E.C. 3.1.1.7) and butyrylcholinesterase (BChE, E.C. 3.1.1.8). Under physiological conditions, AChE terminates the action of acetylcholine at synapses. AChE is also implicated in the differentiation of embryonic stem cells, neuritogenesis, cell adhesion, synaptogenesis, activation of dopamine neurons, amyloid beta fiber assembly, haematopoiesis and thrombopoiesis, or regulation of glutamate-mediated hippocampal activity. Many compounds target to inhibit this enzyme in order to symptomatically counteract low cholinergic tone; however, irreversible AChE blockade may have fatal consequences. This phenomenon is typical for a class of highly toxic compounds—nerve agents and pesticides. The role of BChE is still extensively discussed; it plays an important role in cholinergic mediation, it contributes to neurogenesis, and has a detoxifying effect towards different xenobiotic drugs. It is also assumed that BChE overtakes the function of AChE in the case of malfunction or later stages of Alzheimer’s disease (AD).

Based on the abovementioned, both AChE and BChE are considered as highly relevant targets in the field of medicinal chemistry. For neurodegenerative disorders such as AD, there is a strong consensus that AChE/BChE reversible inhibitors can, at least temporarily, alleviate the symptoms associated with the disorder, and enhance the cognitive performance of individuals. Other cholinesterase ligands, namely cholinesterase reactivators, typically endowed with strong nucleophilic function, can revert the irreversible action of organophosphorus compounds (nerve agents and pesticides). However, there are many other areas of research involving AChE and BChE, for example, pesticides; inflammation; and other neuronal disorders such as Lewy body dementia, Parkinson’s disease, myasthenia gravis, and so on.

The scope of this Special Issue of Biomolecules, “Cholinesterase Research”, was to provide a broad and updated overview of all the aspects that encompass cholinesterase research. The collection includes cholinesterase structural aspects, drug design and development, in vitro biochemical studies, animal studies, and computational approaches, all devoted primarily to cholinesterases.

Computational studies focus mostly on the structural and dynamical aspects of cholinesterases: first of all a comprehensive review of cholinesterase modelling and simulation was provided by De Boer and colleagues [1]. It analyses AChE/BChE structure and function using computer-based modelling and simulation techniques using different models of both enzymes. It also discusses key structural similarities in the active site gorges of the two enzymes, such as flexibility, binding site location, and function, as well as differences, such as gorge volume and binding site residue composition. Catalytic studies are also described, with an emphasis on the mechanism of acetylcholine hydrolysis by each enzyme and novel mutants that increase catalytic efficiency. The review also explores the inhibitory properties of several compounds currently approved by the FDA and other experimental drugs through Monte Carlo-based docking calculations and molecular dynamics simulations.

The study by Jończyk and colleagues [2] explores molecular mechanisms determining the efficiency and selectivity of individual oximes to reactivate AChE/BChE blocked by sarin and tabun. The study investigated the reactivation of AChE and BChE by selected oximes using molecular docking methods. It identified amino acids essential for effective reactivation and those responsible for the selectivity of individual oximes against inhibited AChE/BChE. The observation made herein can significantly contribute to support the search for new effective reactivators.

The paper of Lushchekina et al. [3] investigated the effect of different concentrations of sucrose on the protein and water dynamics in cholinesterases. It revealed a non-linear correlation with increasing sucrose concentration, i.e., first a decrease in the dynamics at 5 wt% followed by a gain at 10 wt% sucrose. The explanation of this phenomenon is that sucrose molecules interact with the surface of the protein and the entrance of the gorge at a lower concentration through the water layer, damping the motions at the surface, but increasing them inside the gorge. When increasing the sucrose concentration more, the sucrose molecules replace some of the water molecules at the surface, permitting again more water molecules to enter the gorge and opening simultaneously new pathways, among them the hypothesized backdoor to the gorge.

The study by Zueva and colleagues [4] is a kinetic study corroborated by molecular modelling simulation of human AChE inhibition by fluorinated acetophenone derivative, namely 1-(3-*tert*-butylphenyl)-2,2,2-trifluoroethanone (TFK). TFK was found to be a competitive type inhibitor reaching steady state inhibition slowly. It is speculated that after binding, TFK acylates the active serine, forming a hemiketal; the disruption of such complex, i.e., deacylation is slow. Modelling of interactions between TFK and AChE active site by QM/MM showed that the “isomerization” step of enzyme-inhibitor complex leads to a complex similar to substrate tetrahedral intermediate, a so-called “transition state analog”, followed by a labile covalent intermediate. TFK can be classified as a slow-binding inhibitor with potential dual effect that could be of interest in palliative therapy of AD or protection of central AChE against organophosphorus compounds.

When it comes to a development of novel chemical compounds targeting cholinestarases both in the inhibitory and reactivation manner, the Special Issue contains the following experimental studies: the article by Konecny et al. [5] describes the design, synthesis and biological evaluation of a series of 15 novel fluoren-9-amine derivatives as dually active cholinesterase inhibitors and *N*-methyl-d-aspartate receptor (NMDAR) antagonists. The study builds on the concept of so-called multi-target directed ligands (MTDLs) that are believed to provide higher benefit compared to single-oriented drugs. The compounds under the study were initially in silico screened for CNS and oral availability, fitting all the prediction models used. Ongoing assessment of the biological profile included determination of the cholinesterase inhibition and NMDA receptor antagonism at the GluN1/GluN2A and GluN1/GluN2B subunits of NMDAR, along with a low cytotoxicity profile in the CHO-K1 cell line. Compounds were found to be highly selective BChE inhibitors with antagonistic activity on the NMDARs.

The group of Florian Nachon compared in vitro and in vivo efficacy, and toxicity of a hybrid tetrahydroacridine pyridinaldoxime reactivator, namely KM297, with pralidoxime [6]. The study revealed that blood–brain barrier crossing capacity of KM297 in vitro exceeds the permeability coefficient of pralidoxime twice. However, KM297 is also endowed with higher cytotoxicity, particularly on bone marrow-derived cells. Its strong cholinesterase inhibition potency seems to be correlated to its low protective efficacy in mice exposed to paraoxon. Ventilatory monitoring of KM297-treated mice by double-chamber plethysmography displayed toxic effects at the selected therapeutic dose.

Natural compounds and their effect on the cholinesterases are involved as well. Namely, the article by Amat-ur-Rasool and colleagues [7] screened methanolic extracts from seven commonly cultivated plants for their nutraceutical potential with particular emphasis on AChE/BChE inhibition and antioxidant capacity. The majority of extracts inhibited AChE and BChE, with henna and eucalyptus extracts highlighted as the most potent ones. Moreover, all plant extracts were able to scavenge free radicals in a concentration-dependent manner, with eucalyptus being the most potent antioxidant.

The article by Al Mamun and colleagues [8] describes the isolation of thirteen known and three previously undescribed alkaloids of belladine structural type. Notably, significant human BChE inhibition was demonstrated by newly described alkaloids carltonine A and carltonine B, representing a new scaffold for generation of a novel structural type of BChE inhibitors with highly selective pattern over AChE.

Finally, the effect of clinically used donepezil has been investigated: the study of Audira and colleagues [9] is dedicated to donepezil, a currently approved drug for mild-to-moderate stages of AD. The study exploits a zebrafish model to analyze potential adverse effects of donepezil on the short-term memory, behavioral and biochemical changes. Donepezil caused a slight improvement in the short-term memory of zebrafish and induced significant elevation in aggressiveness, while the novel tank and shoaling tests revealed anxiolytic-like behavior. The latter can be ascribed to alterations associated with an elevation of oxytocin and a reduction in cortisol levels in the brain. Thus, chronic waterborne exposure to donepezil can severely induce adverse effects on normal zebrafish in a dose-dependent manner.

In another article by Al-Hamed et al. [10], postoperative administration of donepezil on bone healing was studied in the group of Sprague–Dawley rats. After two weeks of donepezil administration, rats were euthanized, and their bones were analyzed by Micro-CT and histology, with the authors concluding that bone defects and implant osseointegration were significantly reduced compared to the saline-treated rats. Histomorphometric analysis pointed to lower immune cell infiltration in bone defects as the possible culprit for disrupted bone healing.

To conclude, this Special Issue describes important findings related to cholinesterases’ physiological and pathological roles, their involvement in metabolic studies, and the influence of different modulators (inhibitors, reactivators) on their activity. All these findings may broaden the knowledge and the impact on clinical and pharmacological applications of different small molecules targeted to AChE/BChE.
